# Principal Connection Between Typical Heart Rate Variability Parameters as Revealed by a Comparative Analysis of Their Heart Rate and Age Dependence

**DOI:** 10.3390/e27080792

**Published:** 2025-07-25

**Authors:** András Búzás, Balázs Sonkodi, András Dér

**Affiliations:** 1 Institute of Biophysics, HUN-REN Biological Research Center, 6701 Szeged, Hungary; buzas.andras@brc.hu; 2 Department of Health and Sports Medicine, Hungarian University of Sports Sciences, 1123 Budapest, Hungary; bsonkodi@gmail.com; 3 Department of Sports Medicine, Semmelweis University, 1122 Budapest, Hungary; 4 Faculty of Health Sciences, Institute of Physiotherapy and Sport Science, University of Pécs, 7624 Pécs, Hungary; 5 Szentágothai Research Centre Physical Activity Research Group, 7624 Pécs, Hungary

**Keywords:** RMSSD, Bland–Altman plot, Parseval theorem, HRV spectral band, autonomic nervous system, entropy and DFA analysis, heart rate dependence, age dependence, correlated and uncorrelated noise, Piezo ion channel

## Abstract

Heart rate (HR) is strongly affected by the autonomic nervous system (ANS), while its spontaneous fluctuations, called heart rate variability (HRV), report about the dynamics of the complex, vegetative regulation of the heart rhythm. Hence, HRV is widely considered an important marker of the ANS effects on the cardiac system, and as such, a crucial diagnostic tool in cardiology. In order to obtain nontrivial results from HRV analysis, it would be desirable to establish exact, universal interrelations between the typical HRV parameters and HR itself. That, however, has not yet been fully accomplished. Hence, our aim was to perform a comparative statistical analysis of ECG recordings from a public database, with a focus on the HR dependence of typical HRV parameters. We revealed their fundamental connections, which were substantiated by basic mathematical considerations, and were experimentally demonstrated via the analysis of 24 h of ECG recordings of more than 200 healthy individuals. The large database allowed us to perform unique age-cohort analyses. We confirmed the HR dependence of typical time-domain parameters, such as RMSSD and SDNN, frequency-domain parameters such as the VLF, LF, and HF components, and nonlinear indices such as sample entropy and DFA exponents. In addition to shedding light on their relationship, we are the first, to our knowledge, to identify a new, diffuse structure in the VHF regime as an important indicator of SNS activity. In addition, the demonstrated age dependence of the HRV parameters gives important new insight into the long-term changes in the ANS regulation of the cardiac system. As a possible molecular physiological mechanism underlying our new findings, we suggest that they are associated with Piezo2 channel function and its age-related degradation. We expect our results to be utilized in HRV analysis related to both medical research and practice.

## 1. Introduction

Heart rate variability (HRV) measures the variation in interbeat intervals from one heartbeat to the next, reflecting changes in the heart rate (HR) over time. Heart rhythm is primarily governed by the synchronized firing of pacemaker cells in the sinoatrial node of the heart muscle, which initiates the cardiac cycle, and the activity of the pacemaker cells is regulated by autonomic efferent neurons and circulating hormones. The complex actions of the underlying interdependent regulatory systems give rise to the variability of the length of the cardiac cycle over different time scales, supporting the optimal performance of the heart under homeostasis. Therefore, HRV is widely considered an important marker of the autonomic nervous system (ANS) [1].

In addition to sympathetic and parasympathetic nervous system (SNS and PNS) activities, respiration and the baroreceptor reflex are considered to influence HRV on time scales ranging from seconds to minutes. In contrast, other ultradian and circadian rhythms driven by factors like metabolism, physical activity, body temperature, and sleep–wake cycles govern HRV over hours to the daily cycle. In this context, it is interesting to note that a recent unpublished preprint paper proposed that Piezo2 ion channel couples the sympathetic and the parasympathetic tone [2] and Piezo2 (and Piezo1) principally drives the fine regulation of the baroreceptors of the circulation and the respiratory system [2,3,4]. In support, earlier research of the Nobel laureate Ardem Patapoutian and his team showed that conditional Piezo2 and Piezo1 gene knock-out mice had baroreflex failure and essentially lost blood pressure and heart rate (HR) regulation [5]. Therefore, this research indeed demonstrated that Piezo ion channels responsible for mechanotransduction have a major role in HR control as well. The aforementioned preprint paper also theorized that Piezo2 is an ultradian sensor while Piezo1 is the diurnal one, and together they contribute to circadian regulation [2].

HRV is widely used in the investigation of the heart, and is considered an essential diagnostic tool in cardiology. Higher HRV is often associated with better fitness and heart adaptability, while reduced HRV is commonly linked to various pathological conditions, including congestive heart failure, diabetic neuropathy, mental disorders, and cancer [6,7].

Researchers and clinicians use time-domain, frequency-domain, and nonlinear indices to measure HRV by typically analyzing time-resolved electrocardiography (ECG) or pulsometry signals [8,9,10]. Time-domain indices are derived from the time series of RR-intervals and capture the variability in HRV over the monitoring period. The mean RR (or NN) interval or, equivalently, the average HR, is the simplest time-domain indicator. Other time-domain measures, such as the standard deviation of normal-to-normal RR intervals (SDNN), represent the overall variability (both short- and long-term), while the root mean square of successive differences (RMSSD) is more suited to quantify short-term variability [8].

Frequency-domain analysis involves calculating the absolute or relative power within high-frequency (HF), low-frequency (LF), very-low-frequency (VLF), and sometimes, for long-term recordings, ultra-low-frequency (ULF) bands [11]. Using a more sophisticated time-frequency analysis tool, the wavelet-transform, Stefanovska and co-workers divided the VLF band into two more intervals, corresponding to distinct spectral peaks found in this range [12]. More generally, it was established that the cardiovascular dynamics (including peaks found in the HRV spectra) can be associated with “deterministic” origins, while there also exists a strong stochastic (noise-like) component [13]. In a series of papers, Stefanovska and co-workers interpreted the “peaks” as a result of coupling among at least five different oscillatory mechanisms (cardiac activity, respiratory activity, myogenic activity, neurogenic activity, and endothelial activity, in descending order of frequency range) [14,15]. Lately, they pointed out that if coupling between oscillations and/or their frequency changes in time, it might lead to a noise-like feature occurrence in conventional spectral analysis [16]. As a possible physiological background, it has recently been raised that mitochondrial high-frequency oscillation converted into low-frequency oscillations by Piezo2 may also be involved in the coupling of the five above-mentioned oscillatory mechanism, involving proprioception, and Piezo2 is likely the principle coupler of these oscillations [2]. It is important to note that in realization of the significance of this coupling mechanism, an expert panel recently redefined respiratory sinus arrhythmia as respiratory heart rate variability [17]. In support, the role of respiration in terms of Piezo2 (and Piezo1 to a lesser degree)-modulated regulation of HRV has been emphasized earlier [3], and the intracranial pressure pulsatility-induced, Piezo2-related coupling of cardio-respiratory and brain rhythmicity was reported as well [18].

Nonlinear methods and indices (e.g., entropy measures such as Shannon entropy, “Forbidden words”, “Plvar10”, or Renormalized entropy of periodograms) assess the complexity and unpredictability of the time series of interbeat intervals [19]. Due to the heart’s complex regulation system, HRV cannot be fully described using linear methods alone, prompting the use of nonlinear techniques [20,21,22,23]. In detail, here, we deal only with the traditionally most frequently used ones, namely, the Poincaré plot, sample entropy (SampEn), and detrended fluctuation analysis (DFA).

Sample entropy SampEn measures the complexity of HRV, while Detrended fluctuation analysis (DFA) evaluates correlations in HRV data across different time scales. Short-term (α1) and long-term (α2) fluctuations are measured by the slopes of a log-log plot of correlation coefficient versus segment length [8,9]. The Poincaré plot visualizes the correlation between consecutive RR intervals by plotting RR_j+1_ against RR_j_, and the shape of the plot is quantified using an ellipse fitted to the data. The ellipse’s width (SD1) and length (SD2) are considered to represent short-term and overall HR variability, respectively [24].

HRV analysis is nowadays routinely applied in clinical cardiology and stress-relaxation studies [25,26,27,28]. When choosing HRV measures from the time-domain, frequency-domain, and nonlinear methods to track clinical progress or performance, the choice often depends on the investigator’s intuition and preference. This choice is further complicated by the well-known but often ignored fact that changes in HR do affect HRV, e.g., a decrease in HR leads to an increase in typical HRV indices, while an increase in HR generally decreases HRV parameters such as SDNN and RMSSD [29,30,31,32]. Nearly all parameters that describe instantaneous HRV (i.e., excluding time-averaged measures) are influenced by HR, such as the time-domain, frequency-domain, or nonlinear measures [33,34,35,36,37], and this dependence is generally pivotal; namely, it usually masks the effects of all the other factors on HRV. For instance, if the relationship between the common HRV parameter RMSSD and the HR is not appropriately considered, then the variability parameter will primarily reflect changes in the heart rhythm, and it will not provide independent physiological information [37].

However, lacking the exact function that describes the relationship between HRV and HR, there has been no practical way to define a single, “heart-rate-corrected” HRV parameter that could properly account for the HR dependence. Some papers even argued that the existence of such a well-defined HRV (HR) function might mean that HRV does not contain extra information to what HR does [34]. Lately, it has been shown that while a well-defined RMSSD (HR) function indeed exists and shows a remarkable invariability on the level of an individual from the hours to months scale (hence it is called the “Master Curve”, MC) [37], momentary deviations from it on the seconds to minutes scale do occur, and reflect changes in the sympatho-vagal balance, due, e.g., to stress or relaxation [38]. Longitudinal analyses also revealed changes in the MC on the decade scale as well [37], which may be associated with the age-dependent degradation of the cardiac regulatory mechanism. Nonetheless, Piezo2 (and Piezo1 to a lesser degree) provides a molecular interpretation for the HR dependence via pressure pulse detection and age dependence through a quad-phasic non-contact injury mechanism arising first as a transient acquired Piezo2 channelopathy, but later chronification follows with a repeated bout of non-contact injuries, leading to the degradation of this fine-tuning protein [4].

As for the HR dependence of the spectral and nonlinear HRV parameters, we should highlight the contribution of Platisa and coworkers, who presented the spectral components of HRV, sample entropy, and DFA exponents as a function of RR intervals [36,39,40,41,42]. For the age dependence of various entropy parameters, Porta et al. and Voss et al. presented valuable data [21,43,44,45].

More recently, Stefanovska and coworkers, as well as Tsaneva and coworkers, presented notable analyses of HRV data by time-and frequency-domain [15,46,47], as well as nonlinear descriptors [10,48,49,50,51].

All the above works, along with many others, are fundamental contributions towards a deeper understanding of the complex mechanism via which the ANS influences the fluctuations of the HR time series; however, a more complete exploration of this stochastic process is needed. Namely, a comprehensive study revealing the connections of the various HRV parameters as a function of HR has still been missing. A justification for such a study is that the HRV curves in this representation are the easiest to interpret in terms of PNS and SNS effects (see, e.g., Figures S2 and S3) on the ultradian time scale of minutes to hours. Age dependence, on the other hand, reflects long-term changes in the cardiac control of ANS, typically on the scale of decades to years. The investigation of such a combination of dependencies, namely both HR and age dependence on a large database, has not been accomplished so far.

Correspondingly, the current study aims to address this gap by performing a comparative statistical analysis of ECG recordings from a public database, containing 24-h-long ECG recordings of 200 healthy individuals, with a focus on the HR dependence of typical time-domain, frequency-domain, and nonlinear HRV parameters, which reflect the same stochastic process from different viewpoints. Furthermore, the large number of recordings and the wide age distribution of the volunteers allowed us to make age-cohort studies as well. In support, the age dependence of the HRV parameters sheds light on the physiological grounds of the complex regulatory mechanism of the ANS on cardiac function, since aging is associated with structural and functional alterations in the brainstem and hypothalamus, regions integral to autonomic regulation [52,53].

Therefore, we propose that our results, clarifying the formal connections between the most typical HRV parameters via analyzing their HR and age dependence, will facilitate a more insightful interpretation of HRV data in cardiovascular research and medical practice.

## 2. Materials and Methods

ECG data were obtained from a public database of the Telemetric- and Holter-ECG Warehouse (“THEW”) at the University of Rochester Medical Center, New York, NY, USA [54]. The 24 h Holter recording data of 202 healthy volunteers (Database Normal, EHOL-03-0202-003, age ranging from 9 to 82 years) were analyzed. In this study, we examined time-domain HRV parameters RMSSD and SDNN directly calculated from the time series of RR intervals extracted from the ECG recordings, the frequency-domain parameters LF, HF, and very-high-frequency (VHF) power obtained from Fast Fourier Transform (FFT) spectra of the RR and dRR time series, while among the nonlinear measures, the DFA exponent α1 (with m = 2 and r = 0.2 × SDNN), and the Poincaré plot were derived from RR time series, according to their definitions, using a MATLAB R2020b code written by one of us (A.B., Supplementary Information). Note that, since here we examined only healthy subjects’ recordings, where the percentage of extrasystoles is negligible, we approximated the NN time series with the corresponding RR time series, filtered for outliers (Figure S1).

The evaluation methods were applied to each valuable ECG recording, and the relationships between the time-, frequency-domain, and nonlinear parameters are shown via HR data of a typical healthy individual (age 21, male), doubled with age-cohort-averaged data of healthy people.

### 2.1. Data Acquisition and Preprocessing

The time series were filtered for outliers by a MATLAB R2020b routine “isoutlier”, moving the median 30 points. Intervals exceeding ±2 s or with biologically implausible HR values (>250 bpm) were excluded. Remaining RR intervals were converted from seconds to milliseconds for further analysis. The cumulative RR time series was also computed to obtain a uniform time base.

### 2.2. Modified Poincaré Analysis and M-Curve Construction

**Figure 1:**
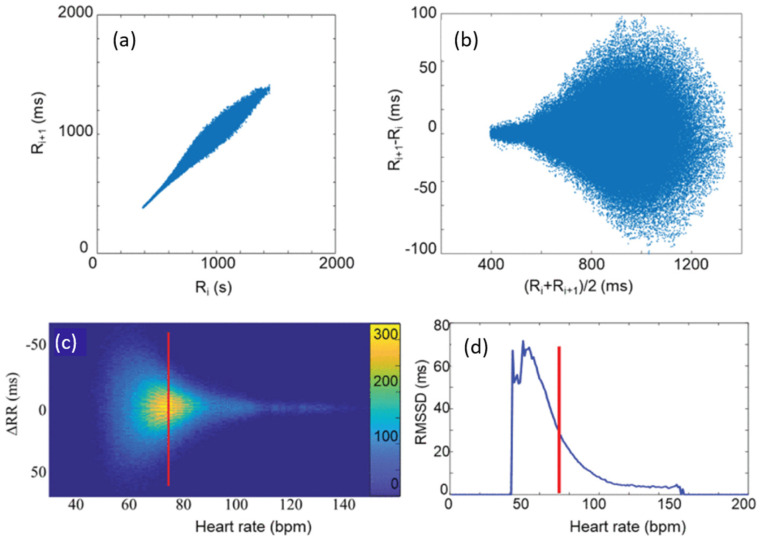
(**a**) Poincaré plot of a typical RR time series. (**b**) The same in Bland–Altman-like representation. (**c**) ∆RR (≡dRR) as a function of heart rate (HR), as calculated from the data in (**b**). The color code shows the frequency of the data. (**d**) The RMSSD versus HR curve (MC), determined from data in (**c**), as the RMS of the distribution of ΔRR values at each HR. The red lines stand for illustration of the way of calculation at an ad hoc HR value. (Reproduced from Búzás et al. [37]).

To characterize the beat-to-beat variability, a modified Poincaré approach was employed. HRV-related Poincaré plots are graphical representations of the relevant RR time series [24]. The Descartian coordinates of the i-th point are R_i_ and R_i+1_, respectively, where R_i_ is the i-th RR interval (Figure 1a). The Poincaré plots can then be transformed to the so-called Bland–Altman representation ((R_i_ + R_i+1_)/2, R_i+1_ − R_i_). Note that the Bland–Altman plot is a difference plot, usually used to compare two different series of measurements of the same thing or process, in order to reveal, e.g., possible systematic differences, due to inaccuracies of the different measuring instruments [55].

Here, we used it in an unusual way, inasmuch as we applied it to the same time series, but one of the counterparts is “shifted” by one data point. Thereby, we arrived at a transformation of the Poincaré scatter plot, where the points are now distributed quasi-symmetrically around the abscissa (Figure 1b,c). From this, after a conversion from the RR- to the HR-scale (Figure 1c), and fitting Gaussians to the dRR distribution at each HR, a high-accuracy RMSSD (HR) plot can be determined, which is called the “Master Curve” (MC) (Figure 1d) [37].

The derivative of the RR intervals (dRR) was computed, and each value was assigned to its corresponding mean RR-derived HR. A two-dimensional histogram was constructed for dRR values across discrete HR bins (1 bpm resolution), resulting in a matrix representation of the variability profile (mPP). For each HR level, a Gaussian model was fit to the dRR histogram to estimate the width of the variability distribution, resulting in the M-curve. Data points were excluded from further analysis if the Gaussian fit quality (R^2^) was below 0.01 or if the data were sparse.

The M-curve was subsequently fit using a two-component noise model detailed in [37]. The model was implemented via nonlinear curve fitting, using the Trust Region algorithm.

### 2.3. Spectral Analysis

The RR and dRR time series were interpolated to a uniform sampling rate of 4 Hz using linear interpolation. Spectral properties were analyzed using a sliding window fast Fourier transform (FFT) approach with a window length of 512 samples corresponding to a time window of 128 s and a step size of 50 samples. In each segment, power spectral density (PSD) was computed for both RR and dRR signals. Note that the length of the epochs we chose was more than two times shorter than what was proposed in a recent paper [20]. The reason for this choice was a trade-off between the resolution of these slow rhythms and the HR change, since we depicted all HRV parameters as a function of HR. Although the ULF and VLF bands were not fully resolved this way, it did not influence our conclusions in the paper.

Standard HRV frequency-domain features were extracted from the PSD. Additionally, the root mean square (RMS) and standard deviation (SD) of each segment were computed. Normalized FFT heatmaps were constructed by averaging the spectral content of RR-derivative segments grouped by HR bins, and then normalizing each bin’s spectrum by its mean spectral profile.

### 2.4. Nonlinear and Complexity Metrics

For each analysis window, the following additional measures were computed:

Sample Entropy (SampEn) using embedding dimension 2 and a tolerance of 0.2 × SD of the signal, DFA α-exponent (DFA α_1_): for the DFA analysis, the time series was divided into non-overlapping segments of lengths ranging from 10 to 100 data points, in 10 steps. These fixed segment sizes (10, 20, …, 100) were used to evaluate the fluctuation function across different time scales. The RR interval time series was interpolated at 4 Hz to achieve uniform sampling. All analyses—including frequency-domain measures (FFT-based spectral analysis), nonlinear metrics (e.g., entropy calculation, detrended fluctuation analysis), and time-domain parameters (e.g., RMSSD, SDNN)—were performed on successive overlapping segments. Each segment contained 512 data points, corresponding to a time window of 128 s. Consecutive segments were shifted by 50 data points (12.5 s), resulting in a considerable overlap between successive windows, which allowed for high temporal resolution in the dynamic analysis.

All computations were implemented in MATLAB R2020b (Supplementary Information), and computed metrics were saved in individual mat files per subject for further group-level statistical analysis.

### 2.5. Age-Cohort Analysis

In order to reveal any systematic dependence of the investigated HRV parameters on the age of the healthy individuals, we performed an age-class cohort study to determine the averaged HRV curves for the following age groups: 0–20 years; 20–40 years; 40–60 years; and 60–80 years. This included data of 73, 86, 32, and 9 volunteers, respectively. In other words, we calculated the mean HRV values of individuals belonging to each age group, at each integer HR value.

**Figure 2:**
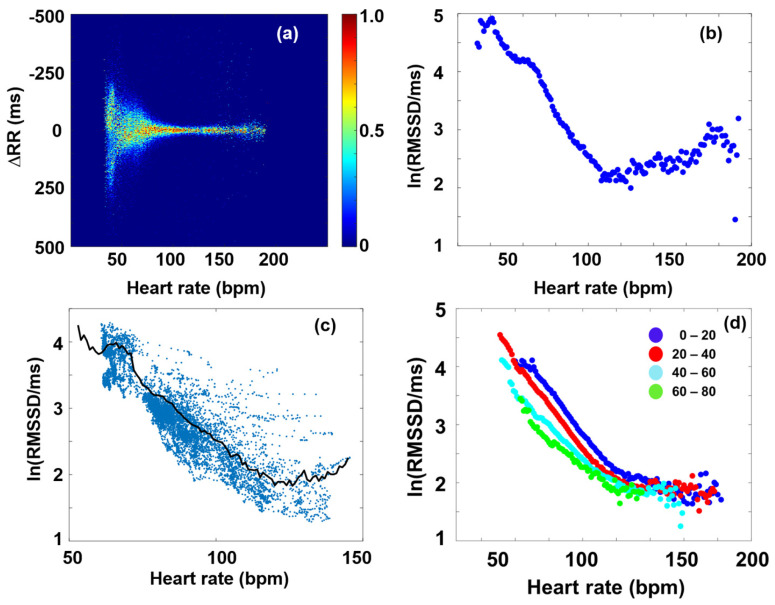
Master Curve plots of a typical individual. (**a**) Bland–Altman plot of RR data, as a function of HR. (**b**) Master Curve (ln(RMSSD versus HR). (**c**) ln(RMSSD) calculated by the conventional method (blue dots) and MC (black line). (**d**) Age cohort-averaged Master Curves, showing an overall decrease in HRV level as age progresses. The different cohorts are distinguished by colors, while their start and end ages in years are indicated aside the symbols.

**Figure 7:**
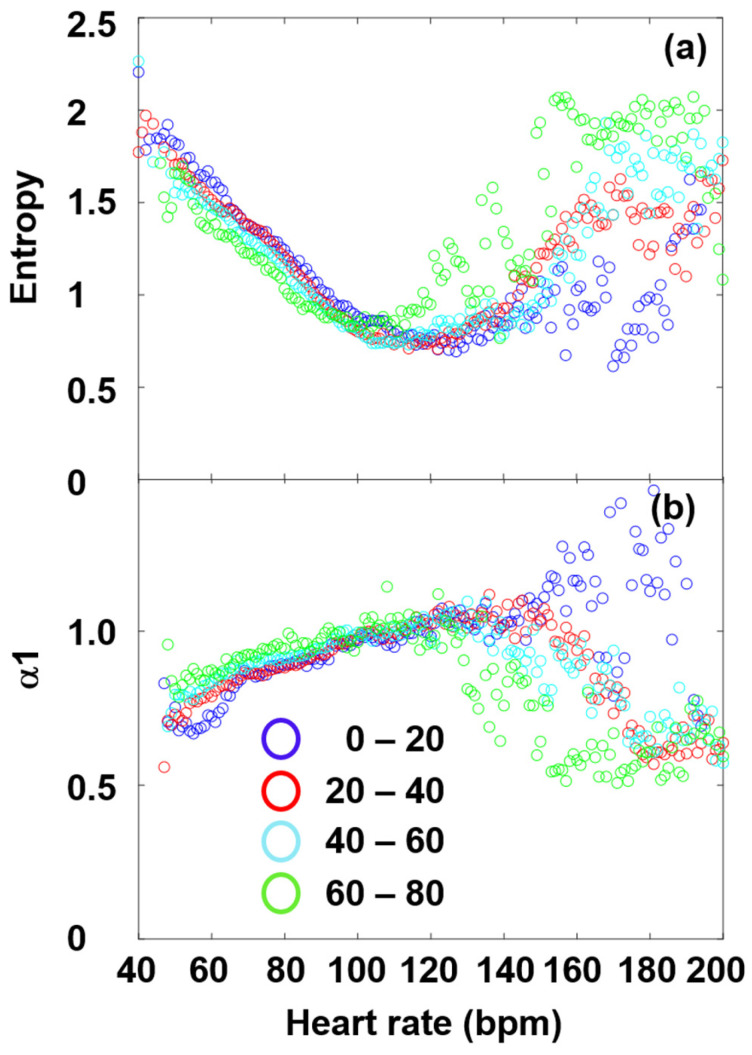
(**a**) Age-cohort-averaged entropy values and (**b**) DFA exponents, as a function of HR.

Notably, the group-averaged M-curves show the characteristic feature of two exponential-like phases separated by a transition zone around a “break point”. The similarly derived curves for the nonlinear measures, SampEn and DFA exponent, however, showed typical “V-shaped” and “dome-shaped” courses, respectively, with turning points close to the break points in the MC. Note that both the M-curves and the nonlinear parameters utilized such measures (Gaussian with of a distribution, entropy, etc.) that are invariant to the sample size, so their respective values for different age cohort can be directly compared, provided that their noise level allows it. In this context, the point-to-point smoothness of the group-averaged curves describing the change in the time-domain and nonlinear parameters versus HR (in terms of relative errors below the break points: 0.0029, 0.0019, 0.0037, 0.0114), and for SampEn (relative errors below the turning points: 0.0117, 0.0032, 0.0096, 0.0144) for the four cohorts in ascending age, respectively), which clearly allowed to reveal the interrelated age dependence of these parameters (Figure 2 and Figure 7), despite that the uncertainty of some of these parameters might be high at some regimes, especially above ca. 150 bpm, for the relatively low numerosity in the eldest cohort group. The definitions of the HRV parameters used in the paper are listed in Table 1.

## 3. Results and Discussion

### 3.1. RMSSD and the Poincaré Plot

Although lately the use of traditional time-domain HRV parameters, such as RMSSD or SDNN, seems to be fading into the background in favor of frequency-domain and nonlinear ones [16,22,45,51], establishing a closer connection between these time-domain and nonlinear measures has recently allowed one to recapitalize the advantages of the former [37]. The new analysis requires several hour-long, preferably 24-h-long, recordings. Figure 2a shows a Bland–Altman-type representation of the conventional Poincaré plot of a typical individual from the THEW data base, where the MC, representing the HR dependence of RMSSD, was calculated, as described above (Figure 2b). Although the nonlinear nature of RMSSD dependence on HR is well-known among experts, the new method based on Bland–Altman plots allowed the determination of the RMSSD (HR) function (called MC) with unprecedented precision, offering a rather sensitive method to measure HRV, which is able to reveal subtle effects, such as those due to delayed-onset muscle soreness (DOMS) [2]. To demonstrate the advantages of the MC method, RMSSD as calculated directly from the RR time series data by a conventional sectional evaluation of the HR time series with a rolling time window (width = 128 s, step size = 12.5 s) is shown together with the MC in Figure 2c. When selecting the optimal width of the time window, one has to consider the problem stemming from the time dependence of HR. If the analysis is restricted to short time intervals (where HR does not change significantly), it results in a higher uncertainty in HRV, as compared to the MC method (Figure 2c). Although longer averages also reduce the level of fluctuations in the sequential time-window analysis, they are inevitably associated with information loss on the HR scale. Hence, whenever it can be applied, the MC method is preferred for the determination of RMSSD (HR) dependence. As established in Buzás et al. [37], the MC is rather stable for an individual on the days-to-months scales, so it can be used as a good reference for the cardiac state of a person. On the cohort level, it was pointed out, however, that aging and myocardial infarction do influence MC, so a change in MC is considered to reflect the remodeling of the heart. In Figure 2d, we show the age-cohort-averaged Master Curves according to a new age classification (the start and end ages of the cohorts are also indicated on the plot), showing a gradual decline of the HRV level, as age progresses [37]. It has also been shown that MCs can be decently interpreted by a two-component stochastic model, where the two noise components were tentatively attributed to sympathetic and parasympathetic influence, PNS effects being more dominant at low heart rates (HR < ca. 100), while SNS effects above (Figure S3) [37]. Recent publications suggest that the MC represents the mean sympatho-vagal balance as a function of HR, and short-term (i.e., minute-scale) deviations from it are indicative of actual fluctuations of the SNS and/or PNS effects [37,38]. A new HRV indicator based on the actual RMSSD normalized to the Master Curve was successfully used to characterize stress responses and relaxation [38].

### 3.2. Fourier Components Versus RMSSD and SDNN

Fourier transform of the RR or dRR time series is used for the spectral description of HRV, commonly using the FFT algorithm. FFT analysis of the RR(t) data usually shows characteristic features in the VLF, LF, and HF ranges, respectively. In the FFT spectra of dRR data, however, the ULF and VLF components are overdamped, and hence, usually only the LF and HF features are considered.

Unless the so-called ULF band (ω < 0.0033 Hz) is investigated, like in the thorough analysis by McClintock and co-workers [15], FFT is usually performed on HRV(t) (i.e., RR or dRR time series) data within a few-minutes-wide time window (of a width usually between 2 and 5 min), which is shifted along the time scale. The RR or dRR time series should be interpolated by equidistant sampling beforehand, and then an average HR value can also be assigned to each interval. Because of the strict mathematical relationship between frequency- and time-domain parameters, if the former shows an HR dependence, the latter should also do so.

**Figure 3:**
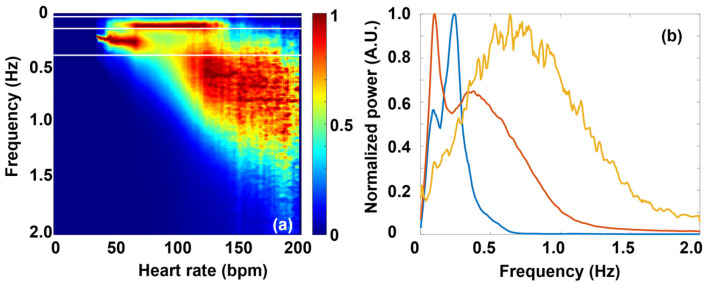
(**a**) Color-coded averaged Fourier map of the dRR data of all the 200 healthy individuals. (The data are normalized at each HR.) The white horizontal lines indicate the border frequencies dividing the different bands (VLF, LF, HF, and VHF). (**b**) Frequency spectra taken at different HRs: 50 bpm (red), 100 bpm (blue), and 150 bpm (yellow).

Figure 3a shows the averaged FFT map of all the healthy people’s dRR data from the THEW database as a function of HR. While the conventional LF and HF bands present explicit spectral features at “normal” heart rates (HR up to ca. 120 bpm), a more diffuse but non-negligible feature also emerges mainly in the VHF region (>0.4 Hz) at higher heart rates (above ca. 100 bpm, Figure 3a,b). One should establish that it appears in the range of high HRs, where SNS effects dominate, and as such, it is expected to be an important status indicator in neurology, cardiology, aging, or sports applications.

Spectral components in the VHF band are rather scarcely discussed in the literature, and their interpretation is still tentative [58]. They were first observed by Akselrod et al. on heart-transplanted patients, and then on healthy subjects, as well [59]. Later on, Chang et al. [60] showed that the VHF components may be influenced by some autonomic maneuvers, such as paced respiration and head-up tilt. More recently, Estévez-Báez et al. investigated the VHF components on healthy subjects and patients with cardiovascular autonomic neuropathy. They tentatively assigned them to the lack of parasympathetic feedback on the heart rhythm [58].

So far, however, no one has investigated the HRV-spectral components in the VHF band systematically as a function of HR. Our Fourier map representation reveals that it is rather a broad spectral feature, typically between 0.3 Hz and 1 Hz, that is also spread along the HR coordinate (hence, we call it “cloud”). It appears only at higher heart rates, typically starting shortly above 100 bpm, just around the breaking point in the MC, where parasympathetic control is assumed to fade away, and sympathetic effects start to dominate. By further raising HR, the VHF cloud increasingly dominates the Fourier map, suggesting its close connection with the SNS effects.

**Figure 4:**
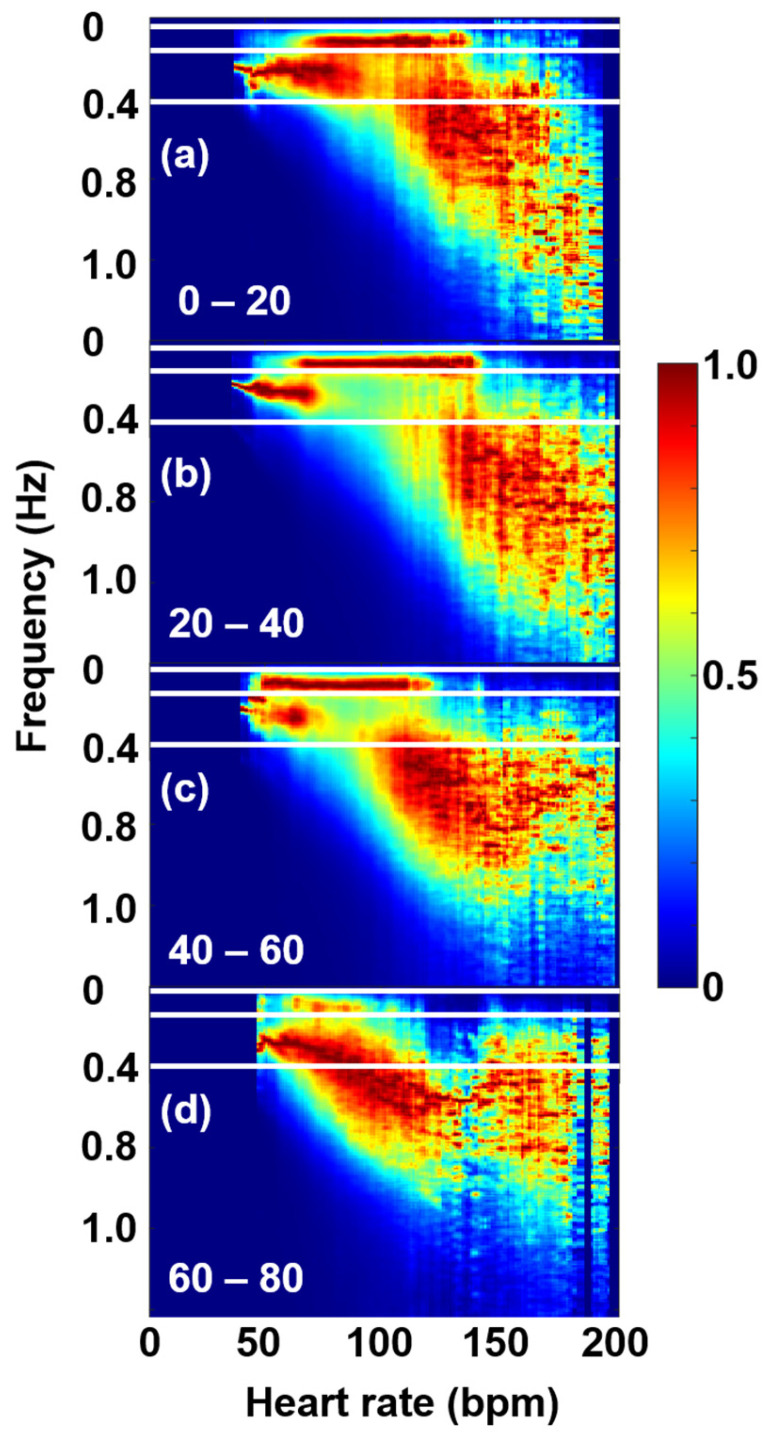
Age-cohort-averaged dRR Fourier maps, showing the shift of the core of the VHF “cloud” to lower HRs. The age cohorts are 0–20 years (**a**), 20–40 years (**b**), 40–60 years (**c**), and 60–80 years (**d**).

Our age-cohort analysis provides interesting support to the above statement: the 24-h-long ECG recordings of the THEW database, registered on healthy people of ages between 9 and 82 years, were classified into five age cohorts, and the cohort-averaged FFT spectral maps were compared (Figure 4).

The position and relative intensity of the cloud shows a marked age dependence, gradually shifting its center towards both lower frequencies and HR values, eventually partially merging into the HF band at ages above 60.

In view of all these, we consider the VHF cloud an important marker of SNS effects of the heart. Although the exact physiological assignment of this feature cannot be made at the moment, a recent study on athletes about the connection of HRV and DOMS may give some hints [2]. Based on these, the current authors interpret this finding as follows: the fine HR-dependent regulation of HRV through Piezo2 (and to a lesser extent by Piezo1) is degraded with the aging process, due to protein degradation. This is in support of the repeated Piezo2 channelopathy-induced quad-phasic non-contact injury model, where the quadric stage is the aging-dependent inflammaging process in association with Piezo2 degradation [4]. Hence, the likely Piezo2-driven coupled modulation between the sympathetic and parasympathetic tone is degraded with aging and pushed to lower HR territories. It is important to note that decoupling is likely driven by GABA, and the Ca_v_1.3 ion channel takes over this HR-dependent regulation of HRV with diminished fine-tuning from there on [2]. However, Piezo2 will likely not be able to convert the high-frequency oscillations of mitochondria to low-frequency oscillations with aging at higher HR domains (not to mention the underlying depletory capacity of mitochondria with aging) due to its loss of the low-frequency Schottky barrier diode-like feature at these higher territories [2]. Therefore, the VHF “cloud” in the HRV representation is suggested by the authors to emerge as a consequence of oscillations of elevated subthreshold calcium current influx-induced calcium wave propagation coupled to Ca_v_1.3 due to Piezo2 degradation, breaking into lower and lower HR territories as age increases. Indeed, Piezo2 is present in the atrium and ventricle of the heart [61,62], and the aging-associated deterioration of calcium handling is observed in human right atrial myocytes [63]. Our interpretation is an in-depth support of earlier observations of VHF. A VHF presence was observed during the exercise of orthotopic cardiac transplanted patients as a likely intrinsic activity of the heart muscle through atrial stretching with no measurable LF component [64,65]. Of note is that LF power is theorized to represent the activity level of Piezo2 [3], and Piezo2 is a stretch-gated ion channel [66]. An important consideration is that Piezo is evolutionarily conserved, and Piezo is the ion channel in charge of mechanical stress buffering by modulation of intracellular calcium handling in Drosophila heart, while the functional mutation of PIEZO fails to mitigate mechanical stress, resulting in pathological remodeling [67].

Further targeted physiological studies are needed to explore the full diagnostic value of this new spectral feature, but it is clear that this effect points at the increased weight of SNS effects, as age progresses. Our new findings are also in line with independent physiological evidence for the increase in the SNS activity by age in humans, which has been observed in both basal (resting) conditions and in response to stress or physiological challenges. In support, Monahan et al. demonstrated that aging is associated with increased sympathetic nervous system activity and reduced parasympathetic modulation, which may contribute to the development of age-related hypertension [53]. Esler et al. observed elevated plasma norepinephrine levels and increased sympathetic nerve firing rates as aging progresses [68].

In support of the reduced parasympathetic modulation, the weight of the HF band shows a marked decrease (until the “VHF cloud” merges into it). This is in line with the findings of Iatsenko et al., who observed a decreased coupling efficiency from the respiratory system to the cardiac one [69].

Due to the strict connection between the HRV(ω) and HRV(t) data (where ω is the angular frequency, and t is time), a quantitative statement can be established for the HR dependence of such time-domain HRV parameters as RMSSD or SDNN and that of the Fourier components of the FFT power spectra. Namely, the following equations hold:

$$SDNN=\frac{1}{N}\sqrt{\sum_{k=1}^{N-1}{\left|X(\omega )\right|}^{2}}\;X(\omega )=F\left\{RR\right\}\;RMSSD=\frac{1}{N}\sqrt{\sum_{k=0}^{N-1}{\left|Y(\omega )\right|}^{2}}=\frac{1}{N}\sqrt{\sum_{k=0}^{N-1}{\omega \left|X(\omega )\right|}^{2}}=\frac{1}{N}\sqrt{\sum_{k=1}^{N-1}{\omega \left|X(\omega )\right|}^{2}}\;\;Y(\omega )=F\left\{dRR\right\}\;\approx \;\omega F\left\{RR\right\}$$

**Figure 5:**
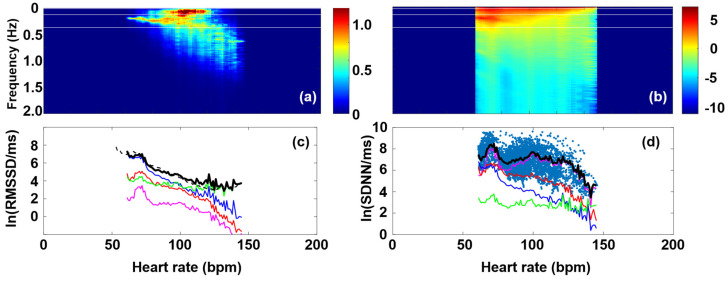
(**a**) dRR Fourier map and (**b**) RR Fourier map of the selected, typical individual. (**c**) Power-spectrum coefficients of the different dRR Fourier-spectral components (VLF (magenta), LF (red), HF (blue), and VHF (green), and their superposition (black), with the almost perfectly overlapping MC (dashed black), as a function of HR. (**d**) Power-spectrum coefficients of the different RR Fourier-spectral components (VLF (magenta), LF (red), HF (blue), and VHF (green), and their superposition (black), with the overlapping scatter plot of the SDNN (HR) values obtained from the conventional (shifting time-window) evaluation (cyan points), as a function of HR.

The name of the underlying mathematical relationship is Parseval’s theorem, which expresses the unitarity of the Fourier transform, loosely stating that the “energy” of the signal should be equal by summing power-per-sample across time or spectral power across frequency [70]. To translate it for our case, the HRV amplitude associated with a certain HR should be the same whether it is calculated from the time-domain parameters or the corresponding frequency-domain ones. From this, it follows that RMSSD (HR) and SDNN (HR) can be regained from the superposition of the LF (HR), HF (HR), and VHF (HR) functions. Using the data of the selected typical individual, Figure 5 shows that it is indeed the case, with good approximation.

### 3.3. Fourier Components Versus Nonlinear Measures (DFA and SampEn)

DFA analysis and SampEn report rather on the structure of HRV fluctuations than on their size. DFA analysis evaluates the degree of correlation between each RR interval and the preceding and following RR intervals across various time scales. The algorithm first divides the integrated time series of 10 intervals into bins of length N. In each bin, a least-squares line is fitted to capture the local trend. The integrated time series is then detrended by subtracting the local trend in each bin. The root-mean-square fluctuation of the integrated and detrended time series is subsequently calculated for all bin lengths, ranging from N = 10 to N = 100 RR intervals. The α coefficient, which quantifies the relationship between fluctuations and bin length on a log-log plot, determines the extent of long-range correlations. An α_1_ value of 1.00 indicates perfect correlations across all time scales, while an α_1_ value of 0.50 signifies complete lack of correlation with previous and subsequent intervals [8,71].

**Figure 6:**
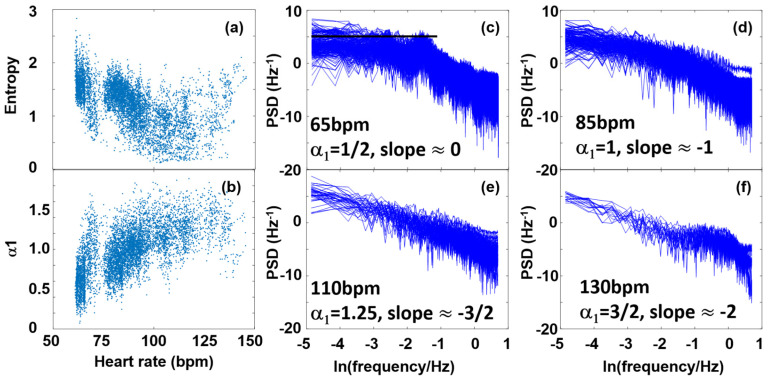
(**a**) SampEn values calculated from RR data in a 120 s-wide sliding window analysis, (**b**) DFA exponents (α1) from a sliding-window analysis. (**c**–**f**) Frequency spectra associated with 65 bpm, 85 bpm, 110 bpm, and 130 bpm, respectively.

Figure 6d shows the α_1_ (HR) plot of the selected individual, whose RR data were used in the former HRV analyses in this paper. In spite of the relatively large standard deviation, the overall trend is clearly visible. While at the lowest HR values (HR ≈ 60–65 bpm), the DFA coefficient, α1 is close to 0.5, there is a rising tendency up to HR ≈ 120–130 bpm, α1 reaching a maximum value of shortly over 1, above which the HR range starts to decline to lower values again. The statistical meaning of this is that while HRV statistics at low HR values are close to that of an uncorrelated white noise, around its zenith it approaches α1 ≈ 1, representing a strongly correlated pink (1/f) noise, and at even higher HR values, it returns toward a less correlated fluctuation statistics. Entropy, reporting the degree of disorder, reflects a corresponding anti-correlation with the DFA exponent. The uncorrelated white-noise-like state corresponds to the highest value, and then, after reaching a minimum showing a stronger level of correlation at HR ≈ 130 bpm, it starts to rise again as HR grows (Figure 6a). Important to note is a recent unpublished preprint paper that assigns this minimum to the Piezo2 inactivation moment [2]. The corresponding frequency spectra are in line with the above: at HR ≈ 65 bpm, the noise spectrum shows a relatively flat feature (slope ≈ 0), while at other HR values it shows a steeper negative slope, reaching its extremum (slope ≈ −2) around 130 bpm, representing a 1/f^2^-like correlated noise.

An age-cohort analysis shows the above relations between the DFA-exponent and SampEn even more clearly (Figure 7). The cohort-averaged plots show a nice anti-symmetry, revealing that the decline of α1 at high HRs is real, whose pendant is seen as a rise of SampEn in the same range. Our findings are in concert with the earlier results of Platisa et al., who depicted the DFA-exponent and SampEn as a function of the RR interval lengths, and found extrema around the same RR value in both cases [39]. They also hypothesized that the abscissa of this “turning point”, H_tp_ (i.e., the minimum place for SampEn and the corresponding maximum place of α1) might reflect the intrinsic heart rate, H_i_ (i.e., the HR measurable at full autonomic blockade) [39,42]. This would mean that H_i_ could be determined non-invasively, without pharmacologically blocking the effects of PNS and SNS on the cardiac cycle. Our age-cohort-averaged data, indeed, show a similar tendency of declination for H_tp_, as it has earlier been determined for H_i_ [72], implying a possible close connection between H_tp_ and H_i_. Note that a decrease in HR_i_ to lower values by age is also in concert with an increasing sympathetic dominance. Recent studies of Singh et al., using other nonlinear HRV measures, such as Approximate Entropy (ApEn) and Recurrence Quantification Analysis (RQA), analyzed two age cohorts (“young” and “elderly” subjects), also came to a similar conclusion [73], suggesting that ApEn and RQA could more sensitively measure age-dependent effects than linear parameters can. It is important to add here that according to earlier literature data, the complexity of HRV also shows a characteristic age dependence, inasmuch as it significantly decreases by age [15,21,43,44,45].

It is also worth emphasizing that similarly to the decrease of H_tp_ and H_i_ values, the center of the VHF “cloud” also decreases as age progresses. This is a nontrivial correlation in the sense that, unlike the weight of the spectral components, the nonlinear HRV parameters do not directly measure the specific peaks considered a manifestation of ANS-related effects, but rather reflect the background noise structure, probably governed by coupled oscillations and fluctuations of other regulatory processes [47]. Hence, they must carry some independent information to those HRV time- or frequency-domain parameters that measure the amplitude (power) of fluctuations.

## 4. Conclusions

Based on the intrinsic HR and age dependence of typical time- and frequency-domain HRV parameters and nonlinear HRV indices, we carried out a comparative study to reveal their fundamental connection, which is substantiated by basic mathematical considerations, and was experimentally demonstrated via the analysis of 24 h of ECG recordings of more than 200 healthy individuals.

We confirmed that the HR dependence of one of the most frequently used time-domain parameters, RMSSD, can be derived from Poincaré plots, and that this “Master-Curve” representation seems to be superior to the conventional method. The MC reflects both PNS and SNS effects, and represents the medium-term sympatho-vagal balance (from the hours to months scale). Momentary deviations from it (on the second to the minute scale) are indicative of the actual changes in, e.g., mood or stress [38], while long-term changes (on the years to decades scale) can be attributed to the irreversible decline of the complex regulatory mechanism governing the dynamics of cardiac function (see [37]). Moreover, we relate both the HR dependence and age dependence to Piezo2 function.

In addition to this, we showed that the RMSSD (HR) and SDNN (HR) plots can also be reproduced from the Fourier-spectral components of the original RR time series, which is a natural manifestation of the Parseval theorem. As an illustrative tool for the visualization of the HRV spectra, we used a novel Fourier map representation. By comparing the HR dependence of the Fourier map and the Master Curve, we found a new cloud-like structure of the HRV frequency spectrum. It is confined mainly in the VHF band, and must be an important indicator of the effects of sympathetic nervous system on the heart rhythm. By an age-cohort evaluation, it was found that this “VHF band” is gradually shifted to lower HR regimes, as age progresses, and as such, it may be an important indicator of the age-related loss of remodeling capacity of the heart at gradually lower HR territories. It is noteworthy that acquired Piezo2 channelopathy is proposed as the breach of homeostatic remodeling [4], or the aforementioned pathological remodeling. We expect it to be utilized in the diagnostics of various cardiovascular diseases in the near future [68].

An anti-correlation between the DFA exponent (α) and sample entropy is apparent both on the individual and the cohort levels, which could be interpreted by frequency-spectral features of the HRV, as well. One can establish that these nonlinear indices report on the stochastic structure of HR fluctuations, contrary to the other time- or frequency-domain HRV measures that reflect direct PNS and SNS effects on the power of the HRV signal, and hence, they carry independent information about the complex regulatory mechanism of the cardiac cycle. More formally, if we unfold the HRV function to a slowly-changing “amplitude” factor and a rapidly oscillating “noise” factor, we can write HRV in the time and frequency domains: HRV(t) = a(t)ˑξ(t), and HRV(ω) = A(ω)*Ξ(ω), respectively, where the second equation is the Fourier-transformed version of the first, and * stands for convolution. In this case, the typical time-domain parameters (such as RMSSD or SDNN) report on a(t), while such nonlinear parameters as SampEn or the DFA exponent report on the noise structure (ξ(t)), so the two types of measures can carry independent information, and hence, for the full description of HRV, both are necessary. The frequency-domain HRV(ω) parameters, on the other hand, are determined by both. Accordingly, the age dependence of the extremum places of these nonlinear measures seems to show correlation with the frequency-shift of the diffuse structure of the HRV spectrum, which can probably be assigned to increasing SNS dominance, as age progresses.

On the whole, we believe that our new findings regarding the phenomenology of HRV will shed more light on the context of various HRV measures, and will contribute to a deeper interpretation of the effects of external and internal factors associated with normal physiological or pathological phenomena.

## Figures and Tables

**Table 1:** List of the main parameters used in this study.

Parameter	Definition	Mathematical Formula
RMSSD	Measures short-term HRV based on the square root of the mean of the N − 1 squared differences between adjacent RR intervals. Reflects parasympathetic activity.	$RMSSD=\sqrt{\frac{1}{N-1}\sum_{1}^{N-1}{\left({RR}_{i+1}-{RR}_{i}\right)}^{2}}$
SDNN	Standard deviation of all RR intervals. Reflects overall HRV (both sympathetic and parasympathetic).	$SDNN=\sqrt{\frac{1}{N-1}\sum_{1}^{N-1}{\left({RR}_{i}-\langle {RR}_{i}\rangle \right)}^{2}}$
VLF (Very Low Frequency)	Power in the very low-frequency range (0.0033–0.04 Hz). Linked to thermoregulation, hormones, and other slow-acting regulatory mechanisms.	$$\int_{0.0033\mathrm{Hz}}^{0.04\mathrm{Hz}}F\left(RR\right)d\omega$$ F$\left(RR\right)$ Fourier transform of RR time series
LF (Low Frequency)	Power in the low-frequency range (0.04–0.15 Hz). Reflects both sympathetic and parasympathetic activity.	$\int_{0.04\mathrm{Hz}}^{0.15\mathrm{Hz}}F\left(RR\right)d\omega$
HF (High Frequency)	Power in the high-frequency range (0.15–0.40 Hz). Mainly reflects parasympathetic (vagal) activity, associated with respiration.	$\int_{0.15\mathrm{Hz}}^{0.4\mathrm{Hz}}F\left(RR\right)d\omega$
VHF (Very High Frequency)	Power in the very high-frequency range (>0.4 Hz). May reflect noise or specific physiological phenomena.	$\int_{0.4\mathrm{Hz}}^{max(\omega )}F\left(RR\right)d\omega$
Entropy (SampEn, ApEn)	Measures the complexity or unpredictability of RR interval time series. Higher entropy = more complex signal [56]	SampEn(m, r, N) = ln(A/B) $A=N\left(d\left[X_{m+1}\left(i\right),X_{m+1}\left(j\right)\right]<r\right)$ $B=N\left(d\left[X_{m}\left(i\right),X_{m}\left(j\right)\right]<r\right)$ N number of vector pairs in our case m=2, r=0.2·SD
DFA (Detrended Fluctuation Analysis)	Detects fractal scaling properties in HRV, capturing long-range correlations. Useful in non-stationary data [57]	$F\left(s\right)={\left[\frac{1}{N_{s}}\sum_{i=1}^{N_{s}}\frac{1}{s}\sum_{j=1}^{s}{\left(y_{i}\left(j\right)-{\hat{y}}_{i}(j)\right)}^{2}\right]}^{1/2}$ $F(s)\propto s^{\alpha }$ α is scaling exponent (s = 10, 20, …, 100)

## Data Availability

The original data presented in the study are openly available in the Telemetric and Holter ECG Warehouse (THEW) at https://doi.org/10.1016/j.jelectrocard.2010.07.015, Ref. [54].

## Supplementary Materials

The following supporting information can be downloaded at: https://www.mdpi.com/article/10.3390/e27080792/s1. Figure S1. Demonstration of the outlier filtering by the MATLAB R2020b routine “isoutlier”, with a moving median of 30 points. Figure S2. Demonstration of the RR recordings and the corresponding HR and RMSSD time series. The bottom inserts depict the log(RMSSD) data in HR and RR representation. Note that in the HR representation, the log(RMSSD (HR)) function looks simpler; namely, it can be approximated by a combination of two linear functions. Figure S3. Similarity between the HR dependences of the two components of our noise parameter (d) and the two components of the autonomous nervous system (ANS), as generally assumed. (adapted from [37]). MATLAB code used for evaluation of raw HR data.

## Abbreviations

The following abbreviations are used in this manuscript:

ANS	Autonomic nervous system
DFA	Detrended fluctuation analysis
ECG	Electrocardiography
FFT	Fast Fourier Transform
HF	High frequency
HR	Heart rate
HRV	Heart rate variability
LF	Low frequency
MC	Master Curve
NN	Normal-to-normal RR intervals (NN and RR are used here synonymously)
PNS	Parasympathetic nervous system
RMSSD	Root mean square of successive differences
RR	Length of the RR interval
dRR	Length-difference in successive RR intervals
SampEn	Sample entropy
SDNN	Standard deviation of normal-to-normal RR intervals
SNS	Sympathetic nervous system
THEW	Telemetric- and Holter-ECG Warehouse
VHF	Very high-frequency
VLF	Very low-frequency
