# Supplementary material for: Principal Connection Between Typical Heart Rate Variability Parameters as Revealed by a Comparative Analysis of Their Heart Rate and Age Dependence
Source: Entropy (Basel). 2025 Jul 25;27(8):792. doi: 10.3390/e27080792 (PMC12385741; doi:10.3390/e27080792)
Supplement: Supplementary file 1 [file entropy-27-00792-s001.zip › entropy-3665828-supplementary.pdf]

Supporting information

András Búzás, Balázs Sonkodi and András Dér

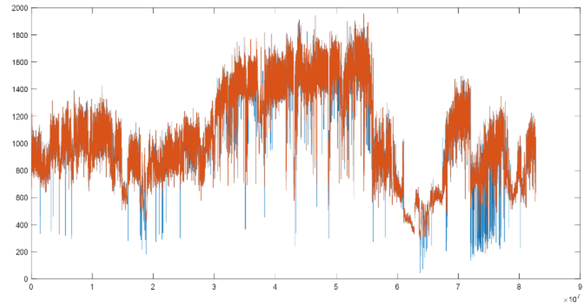

Figure S1. Demonstration of the outlier filtering by the MATLAB routine ”isoutlier”, with a moving median of 30 points.

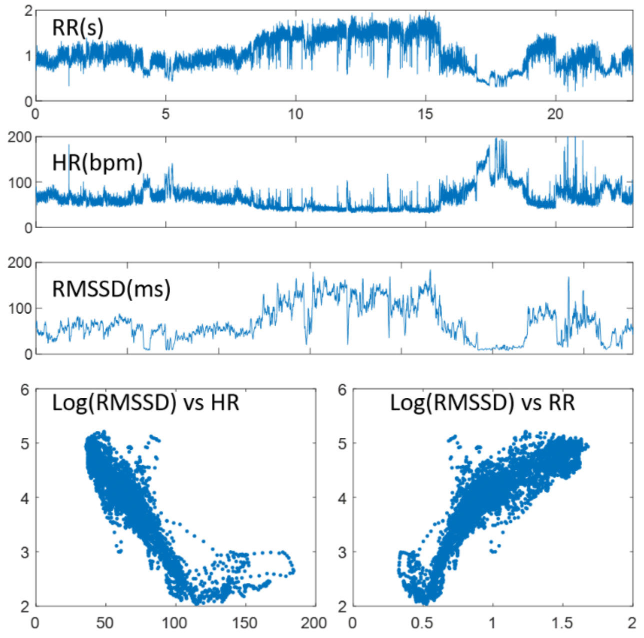

Figure S2. Demonstration of the RR recordings, and the corresponding HR and RMSSD time series on the example of a volunteer. The bottom inserts depict the log(RMSSD) data in HR- and RR.representation. Note that in the HR representation, the log(RMSSD(HR)) function looks simpler, namely, it can be approximated by a combination of two linear functions.

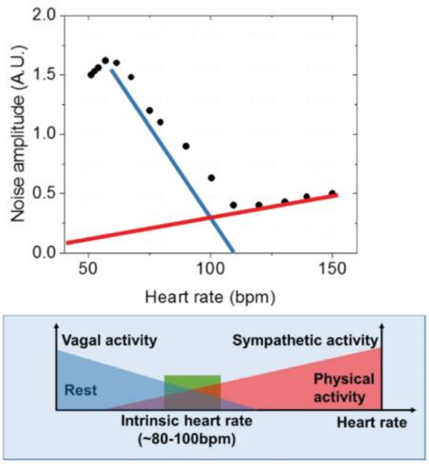

Figure S3. Similarity between the HR-dependences of the two components of our noise parameter (d) and the two components of the autonomous nervous system (ANS), as generally assumed. (adapted from Búzás et al., 2022)

HRV_HR_analysis_commented.m

```
clear
clc

for sel=11
   clc
   clearvars -except sel

   %% Data loading and preparation
   m = 200;
   HRmax = 200;
   MS = 500;
   smth = 1;

   [A, agecorr] = DataOpen;          % Load metadata
   name = num2str(A(:,1));           % Subject IDs
   ND = size(name,1);                % Number of subjects

   % Preallocate matrices for results
   Stotal = zeros(HRmax, ND);
   Stref = zeros(HRmax, ND);
   Stup = zeros(HRmax, ND);
   hrage = zeros(HRmax, ND);
   Age = A(:,2);
   Sm = A(:,3);
   mFall = zeros(220, ND);

   %% Read RR and HR data for the selected subject
   display(sel);
   [RR, HR, NR, age] = readRR(sel, A, smth);
   ind = abs(RR) > 2; RR(ind) = []; HR(ind) = [];
   RR = RR * 1000; RR0 = RR;             % Convert to milliseconds
   cRRdata = RR;
   time=cumsum(cRRdata);

   %% Filter RR intervals
   % Moving median outlier removal
   TF = isoutlier(cRRdata, 'movmedian', 30);
   plot(time, cRRdata); hold on;
   indf = find(~TF);
   plot(time(indf), cRRdata(indf));
   timefilt = time(indf);
   RRfilt = cRRdata(indf)';
```

```
%% Compute modified Poincaré plot and M-curve inputs
fRR = (RRfilt(1:end-1) + RRfilt(2:end)) / 2;   % Mean RR
fdR = diff(RRfilt);                        % RR derivative
fHR = 60000 ./ fRR;                        % HR in bpm
fHR(fHR > 250) = 250;
[MC1, ~, mPP, ~, inddel] = MC_beta(fdR, fHR);  % Generate M-curve

%% Fit M-curve using two-component noise model
[a, b, xb, xs, R2, fy, fys, fyp, xmod1, x, y, dd, ss, pp] = fitNoise_beta2(MC1, 30, 20);
plot(log(MC1), 'b.', 'MarkerSize', 20); hold on;
plot(log(fy), 'r');

% Normalize RR derivative by fitted M-curve values
for i = 1:length(fdR)
   ndRR(i) = fdR(i) ./ fy(round(fHR(i)));
end
fndRR = filloutliers(ndRR, 'clip', 'movmedian', 30);

%% Interpolation and FFT-based spectral analysis
time = cumsum(RRfilt) / 1000;
t0 = min(time); t1 = max(time);
SmpRate = 4;                         % Interpolation rate in Hz
v = t0:1/SmpRate:t1;

% Interpolate RR and derivatives
interpRR = interp1(time, RRfilt, v);
interpdR = interp1(time, [diff(RRfilt); 0], v);
interpHR = interp1(time, 60000 ./ RRfilt, v);

%% Sliding-window FFT analysis
fseq = 512; step = 50;
Nft = floor(length(interpRR)/step);
detrendRR = RRfilt - movmean(RRfilt, fseq/2);
PSD = []; PSDN = [];
clear AA1

for fi = 2:Nft - (fseq/step)
   if mod(fi, 100) == 0; disp(fi); end
   seq0 = (fi - 1) * step + 1;
   seq1 = seq0 + fseq - 1;
   st0 = v(seq0); st1 = v(seq1);
   ind = find(time > st0 & time < st1);

   % Interpolated segments
   fRdata = interpRR(seq0:seq1);
```

```
    fddata = interpdR(seq0:seq1);
    fHdata = interpHR(seq0:seq1);

    [psdr, ufr, lfr, hfr, vfr, tpr, freqsr, rmr, sdr] = FourierRRdata2(fRdata, SmpRate);
    [psd, uft, lft, hft, vft, tpt, freqs, rmt, sdt] = FourierRRdata2(fddata, SmpRate);

    fhr(fi - 1) = mean(fHdata);

    % Save spectral features
    uf(fi - 1) = uft; lf(fi - 1) = lft; hf(fi - 1) = hft;
    vf(fi - 1) = vft; rm(fi - 1) = rmt; sd(fi - 1) = sdt; tp(fi - 1) = sum(psd);
    ufn(fi - 1) = ufr; lfn(fi - 1) = lfr; hfn(fi - 1) = hfr;
    vfn(fi - 1) = vfr; rmn(fi - 1) = rmr; sdn(fi - 1) = sdr; tpn(fi - 1) = sum(psdr);

    PSD = [PSD; psd];
    PSDN = [PSDN; psdr];

    % Complexity and variability metrics
    Eni(fi - 1) = SampEn(detrendRR(ind)', 2, 0.2);
    NdRR(fi - 1) = rms(fndRR(ind));
    rmsRR(fi - 1) = rms(diff(RRfilt(ind)));
    strRR(fi - 1) = std(RRfilt(ind));
    [alpha, ~] = DFA_fun(RRfilt(ind), 10:10:100);
    AA1(fi - 1) = alpha(1);
end

%% Construct normalized FFT map per HR
fftmap = zeros(200, size(PSD,2));
PSD(isinf(PSD)) = NaN;
for fi = 1:200
    indm = find(round(fhr) == fi);
    if ~isempty(indm)
        linem = mean(PSD(indm,:), 'omitnan');
        fftmap(fi,:) = linem ./ mean(linem);
    end
end

%% Final summary plots
subplot(5,4,[1 4]);
plot(timefilt/1000/3600, RRfilt/1000); xlim([0 max(timefilt/1000/3600)]);
subplot(5,4,[5 8]);
plot(timefilt/1000/3600, 60000 ./ RRfilt); xlim([0 max(timefilt/1000/3600)]);
ylim([0 200]); xticklabels({});
subplot(5,4,[9 12]);
plot(rmsRR); xticklabels({}); xlim([0 length(rmsRR)]);
```

```
    subplot(5,4,[13 14 17 18]);
    plot(fhr, log(rmsRR), '.');
    subplot(5,4,[15 16 19 20]);
    plot(60./fhr, log(rmsRR), '.');

    %% Save computed metrics
    savename = [name(sel,:) '.mat'];
    S.age = age;
    S.HR = fhr;
    S.lf = lf; S.hf = hf; S.vf = vf; S.uf = uf; S.tp = tp; S.rm = rm; S.sd = sd; S.PSD = PSD;
    S.lfn = lfn; S.hfn = hfn; S.vfn = vfn; S.ufn = ufn; S.tpn = tpn; S.rmn = rmn; S.sdn = sdn; S.PSDN =
PSDN;
    S.freqs = freqs;
    S.NdRR = NdRR; S.rmssd = rmsRR; S.SDNN = strRR;
    S.En = Eni; S.Alpha = AA1; S.MC = MC1;
    save(savename, 'S');
end



% === MC_beta function ===
% Computes M-curve based on histograms of RR derivatives at each HR level
function [MC1, MC2, mPP, mPPf, inddel] = MC_beta(fdR, fHR)
    fdRmod = fdR + 2.5*rand(length(fdR),1) - 1.25;  % Add noise to smooth histograms
    div = -500:1:500;  % Histogram bins
    mPP = zeros(1001, 250);
    mPPf = zeros(1001, 250);
    MC1 = zeros(250,1);
    MC2 = zeros(250,1);
    x = div;
    inddel = zeros(size(fdRmod));
    inddel(fHR > 250) = 1;

    for hri = 1:250
        inddata = find(round(fHR) == hri);
        data = fdRmod(inddata);

        if length(data) > 3
            y = hist(data, div);
            y = y / max(y);
            gaussEqn = 'a*exp(-((x-b)/c)^2)';
            startPoints = [1 0 150];
            [f1, goodness] = fit(x', y', gaussEqn, 'Start', startPoints);
            R2 = goodness.rsquare;
```

```
        if R2 > 0.01
            mPP(:, hri) = y;
            w = abs(f1.c);
            y2 = y;
            threshold = round(3 * w);
            y2(threshold+501:end) = 0;
            y2(1:501-threshold) = 0;
            mPPf(:, hri) = y2;
            MC1(hri) = sqrt(sum(x.^2 .* y2) / sum(y2));
            MC2(hri) = w / sqrt(2);
            idd = data > threshold | data < -threshold;
            inddel(inddata(idd)) = 1;
        else
            inddel(inddata) = 1;
        end
    else
        inddel(inddata) = 1;
    end
  end
end


% === fitNoise_beta2 function ===
% Fits the M-curve using a double-noise model (slow and fast component separation)
function [a, b, xb, xs, R2, fy, fys, fyp, xmod1, x, y, dd, ss, pp] = fitNoise_beta2(MC1, in1, in2)
  x = 1:250;
  y = MC1;
  ind = y == 0;
  y(ind) = [];
  x(ind) = [];
  indnan = isnan(y);
  y(indnan) = [];
  x(indnan) = [];
  y = y(in1:end-in2);
  x = x(in1:end-in2);

  ft = fittype('dnoise_delta(x,a,b,xb,xs)');

  if size(x,2) > 3
    options = fitoptions(ft);
    options.StartPoint = [0.050 0.05 1.4 0.43];
    options.Lower = [0.020 0 0 0.3];
    options.Upper = [0.300 2 5 1.5];
    [f, goodness, ~] = fit(x', y, ft, options);
    a = f.a; b = f.b; xb = f.xb; xs = f.xs;
```

```
        xmod1 = 1:250;
        fy = dnoise_delta(xmod1, a, b, xb, xs);
        fys = dnoise_delta(xmod1, a, 0, xb, xs);
        fyp = dnoise_delta(xmod1, 0, b, xb, xs);
        [~, dd] = dnoise_omega(xmod1, a, b, xb, xs);
        [~, ss] = dnoise_omega(xmod1, a, 0, xb, xs);
        [~, pp] = dnoise_omega(xmod1, 0, b, xb, xs);

        R2 = goodness.rsquare;
    else
        a = 0; b = 0; xb = 0; xs = 0; R2 = 0;
    end
end


% === FourierRRdata2 function ===
% Computes the power spectral density (PSD) of RR interval signal
% and extracts standard HRV frequency-domain metrics.
% INPUT:
%   data     - RR signal (or its derivative)
%   SmpRate  - interpolation sample rate (Hz)
% OUTPUT:
%   psd      - power spectral density (one-sided)
%   uf       - ultra low frequency power (< 0.04 Hz)
%   lf       - low frequency power (0.04–0.15 Hz)
%   hf       - high frequency power (0.15–0.4 Hz)
%   vf       - very high frequency power (> 0.4 Hz)
%   tp       - total power
%   freqs    - frequency vector
%   rm       - root mean square of input data
%   sd       - standard deviation of input data

function [psd, uf, lf, hf, vf, tp, freqs, rm, sd] = FourierRRdata2(data, SmpRate)
    Nf = length(data);                     % Number of samples
    freqs = SmpRate * (0:(Nf/2)) / Nf;      % Frequency axis (one-sided)
    FRR = fft(data);                       % Compute FFT
    FRR = FRR(1:end/2+1);                  % One-sided FFT
    psd = abs(FRR / Nf).^2;                % Power spectral density


    % Double all components except DC and Nyquist
    if mod(Nf, 2) == 0  % Even length
        psd(2:end-1) = 2 * psd(2:end-1);
    else
```

```
        psd(2:end) = 2 * psd(2:end);
    end

    % Integrate power in standard frequency bands
    uf = sum(psd(freqs < 0.04));
    lf = sum(psd(freqs >= 0.04 & freqs < 0.15));
    hf = sum(psd(freqs >= 0.15 & freqs < 0.4));
    vf = sum(psd(freqs >= 0.4));
    tp = sum(psd);                          % Total power

    rm = rms(data);                         % RMS of original signal
    sd = std(data);                         % Standard deviation
end
```
